# Survey of adolescents’ needs and parents’ views on sexual health in juvenile idiopathic arthritis

**DOI:** 10.1186/s12969-023-00884-x

**Published:** 2023-09-05

**Authors:** Carine Savel, Aurélie Chausset, Pauline Berland, Candy Guiguet-Auclair, Laura Cabane, Bruno Fautrel, Philippe Gaudin, Pascale Guillot, Gilles Hayem, Delphine Lafarge, Etienne Merlin, Nadine Pezière, Christelle Sordet, Sonia Trope, Anne Tournadre, Sandrine Malochet, Jean-David Cohen

**Affiliations:** 1 CRECHE Unit, Department of Pediatrics, INSERM CIC 1405, CHU Clermont-Ferrand, 1 place Lucie & Raymond Aubrac, Clermont-Ferrand, 63100 France; 2 Department of Public Health, CHU Clermont-Ferrand, Clermont-Ferrand, France; 3 Research on Healthcare Performance (RESHAPE), INSERM U1290, Claude Bernard University Lyon 1, Lyon, France; 4 Transversal Patient Education Unit, Department of Public Health, CHU Clermont-Ferrand, Clermont- Ferrand, France; 5 Rheumatology Department, CHU Nice, Nice, France; 6 Service de Rhumatologie, Sorbonne Université, AP-HP. Sorbonne Université, Hôpital Pitié-Salpêtrière, Paris, France; 7 University Grenoble Alpes, T-RAIG, TIMC-IMAG, CNRS UMR 5525, Grenoble, France; 8 Department of Rheumatology, Grenoble Alpes University Hospital, Échirolles, Grenoble, France; 9 Rheumatology Department, Nantes University Hospital, 1 place Alexis Ricordeau, Nantes, France; 10 Rheumatology Department, Paris Saint-Joseph Hospital, Paris, France; 11 Association France Spondyloarthrites (AFS), Tulle, France; 12 Department of Pediatrics, CHU Clermont-Ferrand, Clermont-Ferrand, France; 13 Association Kourir, Paris, France; 14 Department of Rheumatology, National Reference Center for Systemic Autoimmune Diseases (RESO), Hôpitaux Universitaires de Strasbourg, Strasbourg, France; 15 Association Nationale de Défense Contre l’Arthrite Rhumatoïde (ANDAR), Paris, France; 16 Rheumatology, CHU Clermont-Ferrand, Clermont-Ferrand, France; 17 Rheumatology Department, Centre Hospitalier Universitaire de Montpellier, Montpellier, France

**Keywords:** Juvenile idiopathic arthritis, Sexual health, Healthcare providers, Patient education, Adolescents, Parents

## Abstract

**Background:**

Although the advent of new therapeutics for juvenile idiopathic arthritis (JIA) patients has considerably lessened the impact of the disease and reduced its sequelae, the outcomes of JIA remain important in their lives. Disease repercussions and side effects of treatments may affect sexual health and cause psychological distress. This aim of the study was to determine the expectations of adolescent JIA patients and the perceptions of their parents regarding knowledge and communication with healthcare providers (HCPs) in the field of sexual health (SH).

**Methods:**

In France, from September 2021 to April 2022, a survey was conducted, using anonymous self-administered questionnaires, among JIA patients (adults (aged 18–45 years) to provide insights from their recollection of their adolescence) and their parents in nine rheumatology centers and three patient associations.

**Results:**

The responses to the 76 patient questionnaires and 43 parent questionnaires that were collected were analyzed. Half of the patients thought JIA impacted their romantic relationships, but the results were less clear-cut for their sexual activity; and 58.7% of the patients said they would be comfortable discussing the subject with HCPs, but only 26.3% had done so, mainly regarding biomedical issues. The patients and their parents thought that ideally, the topic should be addressed in an individual patient education session at the hospital (51.3% and 34.9%, respectively), in a regular consultation (47.4% and 53.5%), or in a dedicated consultation requested by the adolescent without the adolescent’s parents being informed (38.2% and 20.9%). Most of the respondents thought HCPs should be proactive in SH (77.6% of the patients and 69.8% of their parents). More patients than parents said the following digital information tools must be used: videos (29.0% vs. 9.3%, *p* = 0.0127) and smartphone applications (25.0% vs. 9.3%, *p* = 0.0372).

**Conclusion:**

HCPs should consider addressing the unmet need for SH discussions during their patient encounters. To meet this need, we propose concrete actions in line with the wishes of patients and parents.

**Clinical trial registration number:**

NCT04791189.

**Supplementary Information:**

The online version contains supplementary material available at 10.1186/s12969-023-00884-x.

## Background

Juvenile idiopathic arthritis (JIA) is a group of inflammatory joint disorders that appear before the age of 16 years, last for more than 6 weeks, and have no recognized cause [1]. Its prevalence in Europe and North America ranges from 16 to 150 per 100,000 persons and in France it ranges from 1 to 5 per10,000 persons [2].

Some patients achieve remission after appropriate therapy during their childhood or adolescence. It is estimated that more than 50% of JIA patients will have active disease in adulthood [3]. Although new therapeutics for JIA patients have considerably lessened the impact of the disease and reduced its sequelae [4], the outcomes of JIA remain important in their lives [5–7], particularly, in their sexual activity [8, 9]. Moreover, the unpredictable fluctuations and flares in JIA, which are common in many pediatric chronic diseases (e.g., cystic fibrosis, type 1 diabetes mellitus, asthma, and inflammatory bowel disease), and the difficulties that patients experience in coping with invisible disease (i.e., symptoms associated with the disease may not be externally manifested may thus be harder to detect) [10, 11], may lead to excessive suffering due to the desire to conceal their condition [12]. The management of JIA patients is based on a multidisciplinary care relationship in pediatrics involving three key actors: the patients, their parents, and healthcare providers (HCPs). In France, JIA diagnosis and care are mainly delivered at a hospital, as outpatient care, by multidisciplinary teams, with hospitalization if necessary. In this context, sexuality is especially difficult to discuss [13].

The World Health Organization defines sexual health (SH) as “a state of physical, mental and social well-being in relation to sexuality” [14]. Chronic diseases usually have three levels of impact on the SH of the patients: primary (the disease and its treatments impact the patient’s sexual response) [15–18], secondary (symptoms of the disease) [9, 19–21], and tertiary (related psychosocial impacts) [22, 23].

The disease activity (causing pain, fatigue, and functional limitations) and treatments’ side effects (fatigue, vaginal dryness, and erectile dysfunction), may affect sexual function and lead to psychological distress (low self-esteem and impaired body image) [23–27]. Conversely, one comparative study of young adult women with JIA and healthy controls suggested only a minimal impact. However, this needed to be confirmed by further multicenter studies [28]. Meanwhile, studies on the current understanding and delivery of SH education in rheumatology practices seem scant. An international study has drawn up a list of HCP-identified related topics that need to be addressed by age group [29]: there is clearly interest in SH, the specific SH-related needs of patients and parents have not yet been identified.

In France, the national SH strategy proposes actions for the period 2017–2030 [30]. This strategy is in line with a comprehensive approach to improving sexual and reproductive health. More specifically for JIA patients, actions include investing in SH promotion, especially for young people, addressing the sexuality of people with chronic disease, and promoting research. However, it has been shown that sex education for adolescents and, more broadly, for all young people in schools remains insufficient.

This study determined the expectations of adolescents with JIA and the perceptions of their parents regarding knowledge and communication with HCPs in the field of SH.

## Methods

### Study design and participants

From 24 September 2021 to 8 April 2022, a multicenter survey was conducted among people aged 18–45 years diagnosed with JIA (according to the International League of Associations for Rheumatology classification [31]) and their parents in nine rheumatology centers in France. To optimize the recruitment, three patient and parent associations were also approached: the Association France Spondyloarthrites (AFS, for spondylarthritis patients), the Association Nationale de Défense contre l’Arthrite Rhumatoïde (ANDAR, for rheumatoid arthritis patients), and KOURIR (for parents of children and adolescents with JIA). The participants answered anonymous questionnaires on SH during their adolescence.

Adults (aged 18–45 years) were interviewed to provide insights from their recollection of their adolescence. We chose to interview adults for two main reasons. French law would have required us to obtain prior parental consent to include minors, which might have reduced the response rate, particularly due to the sensitive nature of this subject [32]. In addition, longitudinal studies on SH have shown that adolescent participants might have reported erroneous or contradictory data [32, 33]. Moreover, the findings of a recent study suggested a developmental progression in the robustness and stability of personal memories between adolescence and young adulthood [34].

This research project was approved by the French regional ethics committee (Comité de Protection des Personnes d’Île-de-France IV, No. 2020-A01004-35) and was conducted in accordance with the Declaration of Helsinki.

Two hundred paper survey questionnaires were sent to the rheumatology centers. The process began with a rheumatologist sending all the JIA patients in such centers information on this study and invitations for them to take part in this study. The JIA patients were also asked to extend the invitation to their parents. However, the inclusion of the JIA patients in this study did not require the participation of their parents in the survey or vice versa. In other words, it was not a survey of child–parent pairs. All those who consented to take part in this study (i.e., the JIA patients and their parents) were given two options to answer the anonymous questionnaire: via the (i) paper version or (ii) online version using the REDCap electronic data capture tool [35]. Those who refused to participate in this study were asked to answer another questionnaire that explored such refusals.

In the case of the online questionnaire, the consent statement was included at the beginning of the questionnaire.

The associations were asked to publish on their newsletters and websites an invitation to take part in this study with the link to the online questionnaire and the information leaflet.

Before the questionnaires were designed (one for patients and one for their parents), an extensive literature review was conducted. Then, the pre-final versions of the questionnaires were developed by a panel of experts composed of two sexologists, two rheumatologists, a pediatrician, a therapeutic education nurse sexologist, two biostatisticians, a child psychologist, and the director of a patient association. Two JIA patients and two parents reviewed the pre-final versions for content validity (by removing redundancies, correcting ambiguous questions, and rephrasing items to enhance clarity). Then, the questionnaires were tested on a sample of nine patients and six parents to assess their acceptability.

The participants were also allowed to comment on the questionnaires. The final versions of the questionnaires are presented in English in this paper as supplementary materials: Additional File 1 (for patients) and Additional File 2 (for parents).

### Statistical analysis

A descriptive analysis of the patients’ and parents’ responses was carried out. To ensure that there were no repeat subjects, we cross-referenced the participants’ responses by age, gender, family status, level of education, age of JIA patients at diagnosis, and patients’ JIA subtype. Categorical variables are presented as numbers and percentages, and continuous variables, as means and standard deviations (SDs).

The responses to the open-ended questions were analyzed by AC, a team member with experience in qualitative analysis and CSa, a therapeutic education nurse sexologist involved in the study design, and were classified according to verbatim reports without pre-determined themes.

The responses of the patients and parents were compared using chi-squared tests or Fisher’s exact tests, as appropriate.

All the statistical analyses were performed using SAS® Version 9.4 (SAS Institute, Cary, NC, USA). All the tests were two-sided. Statistical significance was set at *p* < 0.05.

## Results

### Demographic and clinical data

**Fig. 1:**
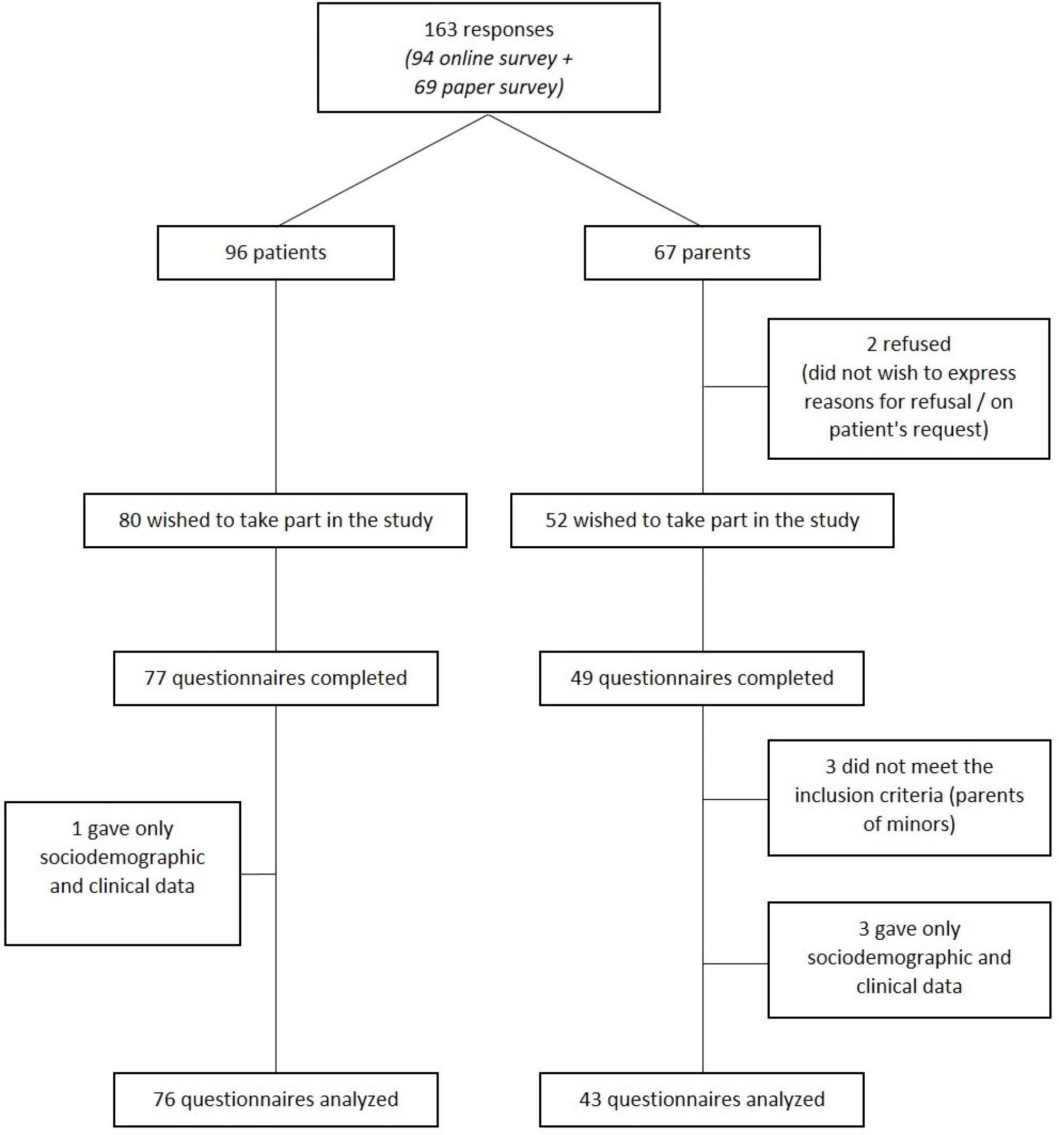
Flow chart of survey participants

For the paper version of the questionnaire, the JIA patients returned 42 out of the 200 distributed questionnaires (response rate: 21.0%), and the parents returned 25 (response rate: at least 12.5%, as we did not know precisely how many patients asked their parents to participate). For the online questionnaires, 35 patients and 24 parents completed them out of the 54 patients and 42 parents who logged on (response rates: 64.8% and 57.1%). Finally, the responses of the 76 patients and 43 parents were analyzed (Fig. 1). The participants’ characteristics are reported in Table 1. Most of the JIA patients were women (75.0%), with a mean age of 25.6 years (SD 7.2) and an educational level higher than high school (89.2%). Their mean age at diagnosis was 8.9 years (SD 5.0). The mean JIA subtype was polyarticular (42.7%) with rheumatoid factor (71.9% of the polyarticular subtype). The mean age of the first sexual intercourse was 18.1 years (SD 3.7), and 64.5% had received a mean of 4 h of sex education in school.

**Table 1:** Characteristics of the participants

	Patients (*N* = 76)	Parents (*N* = 43)
Age (years), mean (SD)	25.6 (7.2)	54.3 (5.6)
Female gender, *n* (%)	57 (75.0)	38 (88.4)
Current family situation, *n* (%)		
Single	27 (36.5)	2 (4.7)
With a partner	46 (62.2)	32 (74.4)
Separated/divorced	1 (1.4)	9 (20.9)
Educational level, *n* (%)		
Primary and secondary school	8 (10.8)	19 (44.2)
Higher-education level	66 (89.2)	24 (55.8)
Parents separated during their adolescence, *n* (%)	21 (30.4)	-
Age of the patients at JIA onset (years), mean (SD)	7.8 (5.0)	7.2 (5.2)
Age of the patients at JIA diagnosis (years), mean (SD)	8.9 (5.0)	8.4 (5.4)
Patients’ JIA subtype, *n* (%)		
oJIA	3 (4.0)	7 (16.3)
pJIA	32 (42.7)	13 (30.2)
ERA	13 (17.3)	5 (11.6)
sJIA	15 (20.0)	6 (14.0)
psoJIA	4 (5.3)	4 (9.3)
undJIA	6 (8.0)	3 (7.0)
unknown	2 (2.7)	5 (11.6)
Patients belonging to a patient association, *n* (%)	15 (19.7)	15 (35.7)

oJIA: oligoarticular juvenile idiopathic arthritis; pJIA: polyarticular juvenile idiopathic arthritis; ERA: enthesitis-related arthritis; sJIA: systemic juvenile idiopathic arthritis; psoJIA: psoriatic arthritis; undJIA: undifferentiated juvenile idiopathic arthritis; HCP: healthcare provider

Most of the parents who participated were mothers (88.4%) with a mean age of 54.3 years (SD 5.6) and an educational level higher than high school (55.8%). They were mainly parents of daughters (81.4%) with a mean age of 24.2 years (SD 5.1). Here again, the JIA diagnosis was prepuberty (mean age 8.4 years, SD 5.4). The most common JIA subtype was polyarticular (30.2%) with rheumatoid factor (for 84.6% of the polyarticular subtype).

### Impact of juvenile idiopathic arthritis on sexual health

Half of the patients thought their rheumatism had an impact on their romantic relationships, as did 51.2% of the parents. The main consequences that the patients mentioned were body shame [46.1% vs. 34.9% for the parents, difference not significant (NS)], low self-esteem (39.5% vs. 20.9% for the parents, *p* = 0.0384), and the fact that the JIA patients felt more mature than their peers (31.6% vs. 18.6% for the parents, NS), which shifted their inter-individual relationships.

For the patients, the impact of the disease on their sexual activity was not clear-cut; 32.9% of them and 23.3% of the parents thought that JIA had affected patients’ sexual activity, mostly due to fatigue (70.6% for the patients vs. 50.0% for the parents) and pain (61.8% for the patients vs. 64.3% for the parents).

52% of the patients reported that they had discussed their romantic relationships with their parents, and 72% of the parents reported having discussed this topic with their child. The parents’ failure to discuss the subject with their child was mainly because it was too emotionally difficult (41.7%), or they lacked knowledge (16.7%).

Participation in a dialogue on their sexual activity was reported by 19.7% of the patients and 55.8% of the parents.

Half of the parents had emotional difficulty in talking about sexual activity with their children, and 11.1% found such discussion culturally complicated, aside from which their child was sometimes closed to dialogue (“Sometimes, he agreed, and sometimes, he didn’t want to talk about it” and “My child refused to talk about his intimacy”).

Of the patients, 58.7% said they would not be embarrassed to discuss SH with an HCP, although only 26.3% reported having done so due to embarrassment, shyness, or lack of self-confidence (41.9%); the subject’s intimacy (19.4%); the attitude or representations of the HCP (16.1%); the presence of their parents (12.9%); and their doubts about the relevance of the subject (12.9%).

Both the patients and the parents discussed SH mainly with a proactive HCP (55.6% and 42.1%, respectively) and with a hospital rheumatologist (50.0% and 68.4%). The main issues discussed were on reproductive health.

Their main sources of information were reference persons in school (46.1%) or in their families (43.4%), although their social networks (i.e., Facebook, Twitter, Wikipedia, and discussion forums) also played a significant role (34.2%).

Nearly half of the parents (45.2%) declared that their child had talked with an HCP, more than what the JIA patients themselves declared (26.3%, *p* = 0.0439). Some of the patients’ and parents’ answers concerning their sources of information significantly differed. While the parents believed that adolescents interacted with HCPs (39.5% vs. 21.1% of the patients, *p* = 0.0305), the young people identified their resources as digital media such as TV programs (21.1% of the patients vs. 2.3% of the parents, *p* = 0.0050); radio (18.4% vs. 0%, *p* = 0.0027); films (18.4% vs. 4.7%, *p* = 0.0344); or magazines (15.8% vs. 2.3%, *p* = 0.0302) as resources.

We note that 27.9% of the parents said they had no source of information on SH. Those of them who reported having had some sources of information on SH cited HCPs most often (30.2%). Most of the parents thought there were few opportunities for dialogue at the hospital (85.4%), as did the patients (92.0%); but 82.9% of the parents and 62.7% of the patients thought SH was important to discuss during their hospital follow-up.

### Patients’ and parents’ needs regarding sexual health

**Fig. 2:**
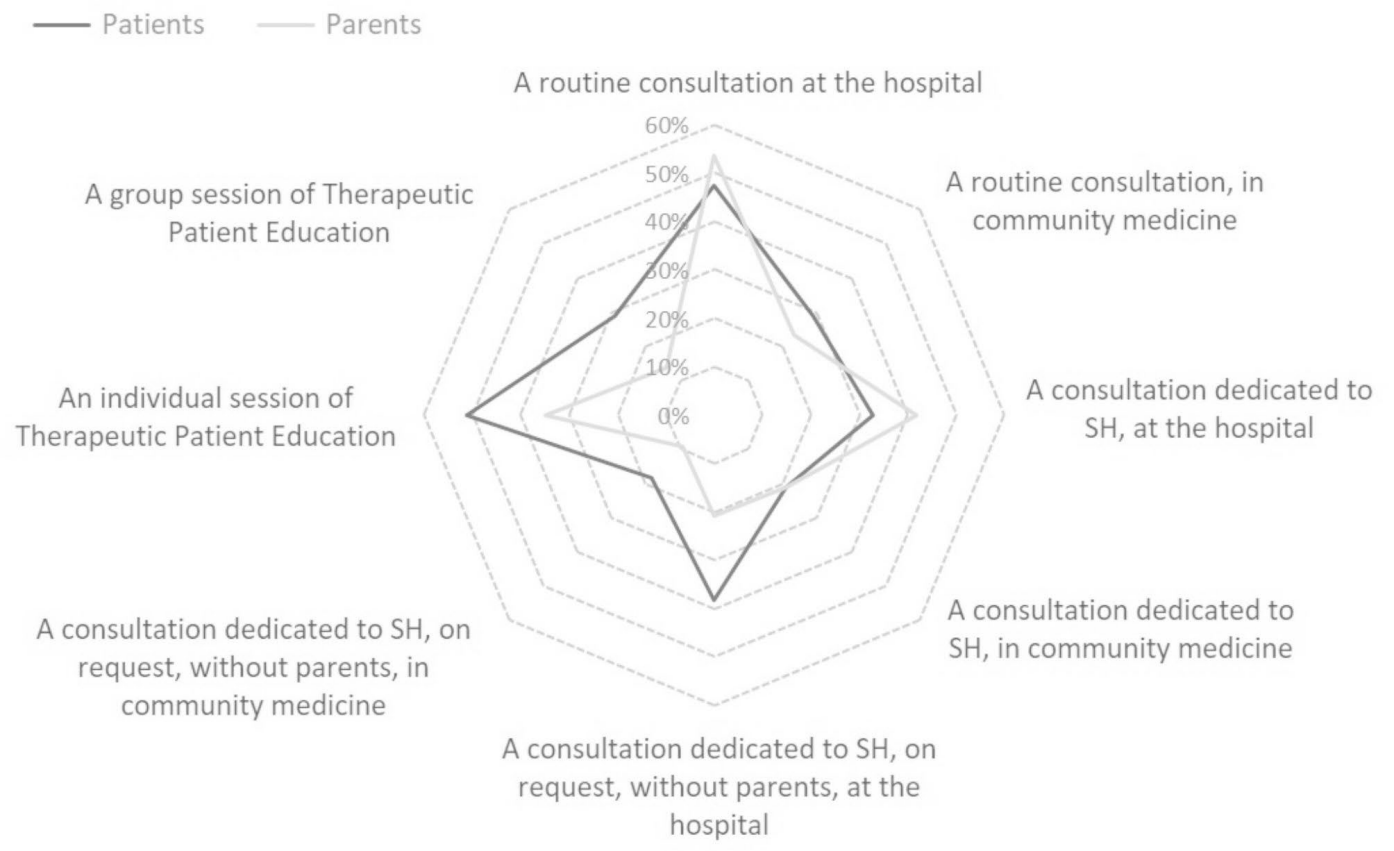
Supports best suited to addressing SH according to juvenile idiopathic arthritis patients and parents. SH: sexual health

Figure 2 presents the needs that the patients and parents expressed in the context of optimal care provision. The results suggested a preference for addressing the subject at the hospital rather than in the community medicine: during an individual patient education session (51.3% for the patients vs. 34.9% for the parents, NS), through regular consultation (47.4% vs. 53.5%, NS), through a dedicated consultation at the adolescent’s request without the parents being informed (38.2% vs. 20.9%, 0.0528), or through a consultation dedicated to this topic (32.9% vs. 41.9%, NS).

### At the hospital

**Fig. 3:**
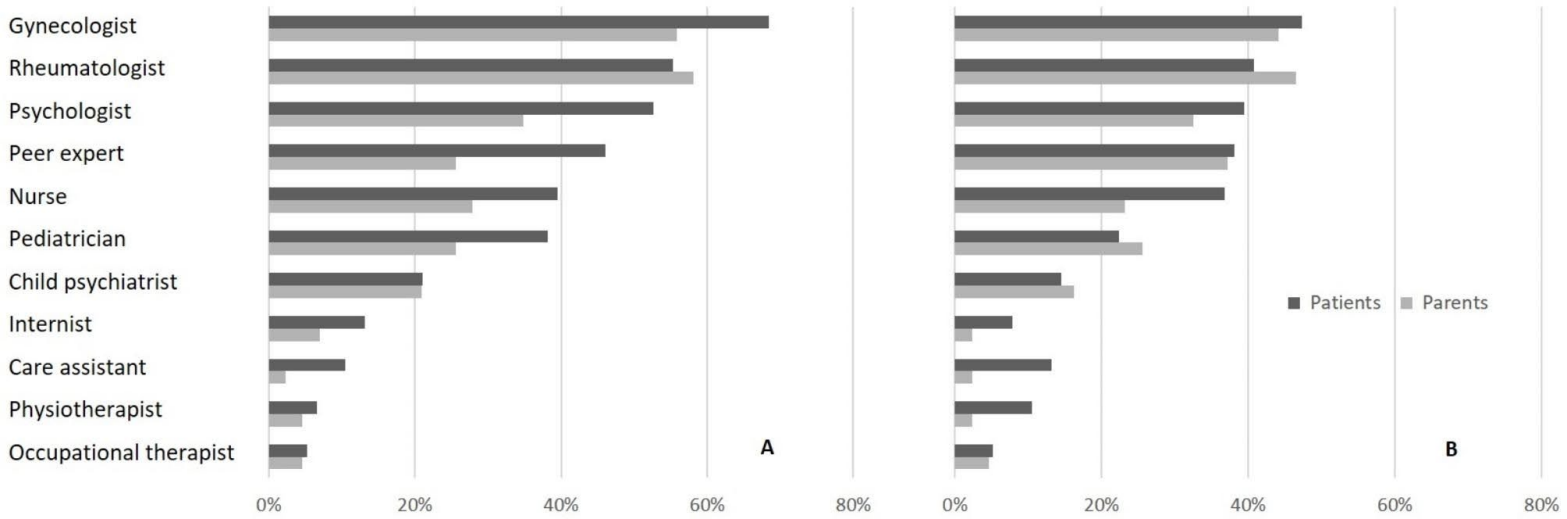
The most competent HCPs (A) and the most approachable HCPs (B) to address SH with juvenile idiopathic arthritis adolescents at the hospital, according to patients and parents. HCP: healthcare provider; SH: sexual health

Both the patients and parents regarded the person most competent to discuss SH as a gynecologist (68.4% and. 55.8% respectively, NS); a rheumatologist (55.3% and. 58.1%, NS); a psychologist (52.6% and 34.9%, NS); or a peer expert (46.1% and 25.6%, *p* = 0.0276) (Fig. 3). They regarded the most approachable persons to discuss SH as a gynecologist (47.4% and 44.2% respectively, NS), a rheumatologist (40.8% and 46.5%, NS), a psychologist (39.5% and 32.6%, NS), or a peer expert (38.2% and 37.2%, NS) (Fig. 3).

### In community medicine

Patients considered a gynecologist (71.1% vs. 48.8% of the parents, *p* = 0.0159), a sexologist (59.2% vs. 13.9%, *p* < 0.0001), a school nurse (42.1% vs. 16.28%, *p* = 0.0039), a family planning counselor (42.1% vs. 13.9%, *p* = 0.0016), and a psychologist (42.1% vs. 37.2%, NS) the most competent to discuss SH in a community. However, the patients were more comfortable with a gynecologist (50.0% vs. 44.2% of the parents, NS), a sexologist (40.8% vs. 14.0%, *p* = 0.0024), a family planning counselor (34.2% vs. 11.6%, *p* = 0.0070), and a psychologist (31.6% vs. 32.6%, NS).

### Communication facilitators

We asked the participants what made it easier for them to discuss SH. The results are reported in Table 2.

**Table 2:** Facilitating items needed to discuss SH with HCPs according to juvenile idiopathic arthritis patients and parents

Facilitating items	Patients (*N* = 76) *n* (%)	Parents (*N* = 43) *n* (%)	*p*
The HCP addressing the issue first	59 (77.6)	30 (69.8)	0.3426
Parents not present at the consultation	51 (67.1)	21 (48.8)	**0.0502**
The “right” time (when questions arise about the patient’s need)	49 (64.5)	23 (53.5)	0.2389
An HCP who is comfortable with the subject	45 (59.2)	23 (53.5)	0.5446
Availability of a brochure on the subject	34 (44.7)	21 (48.8)	0.6665
Discussion of the topic with an HCP of the same gender	34 (44.7)	3 (7.0)	**< 0.0001**
That the HCP identifies the “right” time	24 (31.6)	18 (41.9)	0.2595
Informative video on the subject (viewable by the adolescent)	22 (28.9)	4 (9.3)	**0.0127**
A smartphone application (for information, disease monitoring, etc.)	19 (25.0)	4 (9.3)	**0.0372**
Having time	18 (23.7)	7 (16.3)	0.3408
Being able to speak about it anonymously (via an internet connection)	16 (21.0)	3 (7.0)	**0.0440**
An informal exchange with another adolescent (e.g., while waiting for a consultation or in a hospital room)	12 (15.8)	3 (7.0)	0.1641

SH: sexual health; HCP: healthcare provider; JIA: juvenile idiopathic arthritis

Most of the patients and parents thought that HCPs should be proactive by initiating discussion of SH (77.6% of the patients and 69.8% of the parents).

Regarding the factors that may encourage discussion of SH, those that showed statistically significant differences between the patients and the parents were the presence of parents during such a consultation (67.1% of the patients vs. 48.8% of the parents, *p* = 0.0502) and the HCPs being of the same gender as the adolescent (44.7% vs. 7.0%, *p* < 0.0001). Also, the patients cited the use of digital resources for information significantly more often cited than did the parents: through videos (29.0% vs. 9.3%, *p* = 0.0127) and smartphone applications (including for disease monitoring, etc.) (25.0% vs. 9.3%, *p* = 0.0372).

Finally, 79.0% of the patients wanted to receive biomedical information (e.g., on the teratogenic nature of medications, contraception, the impact of the disease on sexual function, and the medications used to treat sexual dysfunctions), and 68.4% of them wanted to discuss such information, but the parents wanted both less (58.1%, *p* = 0.0158 for biomedical information and 39.5%, *p* = 0.0022 for discussion). Both the patients and parents also wanted reassurance (64.5% of the patients vs. 60.5% of the parents, NS) and to be listened to (51.3% vs. 48.8%, NS) but less frequently mentioned sexual treatment (27.6% vs. 14.0, NS) and being directed to SH-competent professionals (39.5% vs. 27.9%, NS).

All the respondents were interested in the major SH themes (e.g. fertility and rheumatism, pregnancy and rheumatism, how sexuality works (desire, lubrication/erection, orgasm), impact of rheumatism on sexuality, what is sexually normal, side effects of treatments for sexuality, how to treat sexual problems, body image and chronic illness, learning how to communicate with a partner, sexual violence – vulnerability – consent, local services and providers for sexual health, etc.), but those were rarely or never addressed.

## Discussion

To our knowledge, this is the first study that identifies the needs for care in SH of adolescents with JIA and considers the point of view of their parents.

The results provide evidence that this study is relevant, as about half of both the patients and the parents stated having difficulties in romantic relationships due to JIA. Although we did not collect any information on the patients’ global assessment, these difficulties could have been linked to the disease activity [36].

Romantic difficulties in adolescence are real, since sentimental attraction develops from a very young age. In this study, almost half of the patients experienced JIA related body shame and low self-esteem in their romantic relationships. In the same line, a previous study reported emotional difficulties among children and adolescents growing up with JIA, leading to low self-esteem and a distorted body image compared to healthy subjects [37]. Moreover, a meta-analysis found that the presence of a chronic illness influenced teenagers’ self-images [38]. In this study, however, the impact of JIA on sexual activity was less clear-cut. Although almost one-third of the patients had difficulties such as fatigue and pain that decreased their sexual desire and almost two-thirds of these ascribed such difficulties to JIA, the patients could not easily distinguish between difficulties in genital sexual activity because it was their first experience of such and the real impact of the disease on their sexual response. This could explain why they ultimately felt that the impact of the disease on their sexual activity was moderate.

Finally, concerning an overall approach to SH, the patients and the parents had difficulty in separating the emotional, psychosocial, and mechanical aspects of sexuality. For the patients, all the various SH topics (on the biomedical as well as psychological and social aspects) were interesting. Approximately three-quarters of the patients and the parents wanted HCPs to be proactive on the subject. Recent studies on rheumatologic disease in young adults showed similar results. One exploratory study highlighted the critical need for young women and their families to discuss reproductive health issues with their rheumatology teams [39], and another study reported that although men contextualized their parenting challenges and sexual dysfunction as related to their rheumatic disease, they rarely broached the subject with their rheumatologist, and rheumatologists rarely initiated these discussions [40]. In yet another study, nearly two-thirds of the patients thought it was important to raise the subject during adolescence, but only one-fourth felt able to address the issue. Even in the contemporary era of patient education, HCPs are still uncomfortable with addressing the subject [41].

While we were looking for similar studies on other chronic diseases among adolescents and young adults (e.g., asthma, diabetes, inflammatory bowel disease, and cancer), we found a qualitative study on SH among adolescent girls with cystic fibrosis and their parents that was conducted in 2018 in the United States [42] and a cross-sectional survey of adolescents with cancer and HCPs in the Netherlands in 2020 [43]. Their findings are in line with ours: most of the subjects reported that they had never discussed SH, including the impact of their disease and treatment on it, in the context of care, and there were parental barriers to talking about it with adolescents, which were identified as lack of knowledge, a difficult emotional context, and cultural barriers. Moreover, in these two studies, the young people stated that they were interested in digital resources (i.e., videos and smartphone applications) and in talking with their peers to gain a sense of normality, whereas in our study, the JIA patients and their parents significantly differed in their preference for using digital information resources, possibly because of generational evolution. An interesting finding of the study on cystic fibrosis is how the young people accessed the digital resources: they preferred to deal with SH on a general adolescent SH website and then, focus on specific features of the disease to make the pertinent issues visible to the entire adolescent population [42]. This would enable adolescents without chronic diseases to better understand the experiences and constraints of illness and treatment in the contemporary era of mediating consent and promoting empathy in sex education [44]. Following the example of the educational series for teenagers “Sex Education” on the Netflix platform, which addresses topics such as sexual orientation and sexual dysfunction, we could envisage including protagonists with chronic disease (visible or not) who experience positive outcomes in their romantic relationships and sexual activity [45]. It would also be relevant to include testimonies from adolescents with chronic diseases in existing programs like those supported by the Population Council (an international, non-profit, non-governmental organization) [46] and Santé Publique France (a national agency under the Ministry of Health) [47, 48].

The promotion of scientifically validated sites adapted to adolescents could reassure parents who may not see digital tools and assistance from a peer expert as useful sources of information. Online resources would also fulfill adolescents’ wish to access information without their parents being informed and to ask questions anonymously. In fact, some of these materials are already appearing very gradually in sex education media [48]. Digital materials designed for adolescents do seem to be useful learning vehicles for the promotion of overall health and particularly, SH [49–52].

School-based workers, such as nurses, should also be aware of SH issues among adolescents with chronic diseases and of the available related resources [53].

However, digital resources alone cannot meet all the related needs of adolescents and their parents. Aside from being able to access general information on the disease, young people need to discuss them, be reassured, and be listened to.

The participants indicated that they preferred this process at the hospital. However, although the disease seemed to have more strongly affected the adolescents’ romantic relationships than their sexual activity, the HCPs focused on the reproductive health. We can also assume that pediatric rheumatologists are reassuring figures for both JIA patients and their parents, as they are likely to view such persons as experts on the disease, and they would already have established a relationship with one such expert over time. However, these HCPs did not seem sufficiently proactive about the issue. Possible reasons for this are a lack of training, a mismatch between the needs and availability of HCPs involved in SH, and difficulty in broaching this subject with a young person whom they might have watched grow up. Sometimes, the adolescents themselves may be too shy to discuss this issue. Religious convictions or cultural habitus may also hinder the discussion of such a subject. Moreover, the presence of parents can limit adolescent discussion of SH, even though transition recommendations encourage HCPs to carry out adolescent consultations without parents [29].

A previous study that we uncovered on the SH needs of chronic inflammatory rheumatism patients [41] will enable us to target the training needs of HCPs and can help us propose a course specifically adapted to this issue [54].

We noted in this study the confidence of more than half of the patients in sexologists in outpatient practice. The presence of a sexologist at the hospital would allow a patient to transition to such a professional from another professional who had approached the subject but had not been able to go further. Although parents today do not seem to give this specialty much credit, a change in mentality is likely, as today’s children are tomorrow’s parents.

This study had some limitations—mainly, the overrepresentation of persons with higher levels of education and compared to the general population of the same age in France [55]. We can assume that persons with higher educational levels have stronger comprehension and communication skills and thus, would be more inclined to answer a long questionnaire. The overrepresentation of women in this study is not surprising, as they often constitute the majority of adolescents who have most subtypes of JIA and, particularly, the more severe forms that may require adult management (especially the polyarticular form with rheumatoid factor) [3]. Moreover, it is already known that more highly educated women tend to participate more actively in health surveys [56, 57]. The fact that most mothers of women with JIA responded may be because they were more comfortable with telling their mothers than their fathers about this study on such an intimate subject.

Finally, for studies on sensitive issues, such as sexuality, participation rates may be lower [58]. However, the higher level of confidentiality ensured by the anonymous filling out of the questionnaire used in this study provided the subjects a real opportunity to reveal truthful information [59]. The lower response rate of the parents could be explained by the method of distribution (the patients had to ask their parents to take part in this study). Moreover, the lower response rate of the parents could have been caused by a generational barrier to SH discussion, as SH only recently gained momentum and exposure in the media.

## Conclusion

This study highlighted the importance of addressing the SH needs of adolescents with JIA and the need for effective communication between them, their parents, and HCPs in this context. Indeed, in this study, the patients and parents had related expectations that were directly linked to the specific features of the disease. HCPs should consider addressing this unmet need of JIA patients and their parents for SH discussion during their patient encounters, while considering the cultural beliefs and openness of the patients and their parents to sexual education. Thus, both patients and parents should be informed before these conversations on SH occur.

From the results of this study, we suggest the following practical steps in addressing SH as a key problem of adolescents with JIA and other chronic diseases:

- Incorporate information on SH specifics of adolescents with chronic disease into accredited SH education websites and school-based sex education programs;
- Train hospital HCPs and school nurses in SH to enable them to be proactive on the issue;
- Integrate a peer expert into patient education offers;
- Have a sexologist in hospital facilities; and.
- Educate parents about the resources available to their children with JIA and other chronic diseases.

### Electronic supplementary material

Below is the link to the electronic supplementary material.

Supplementary Material 1

Supplementary Material 2

## Data Availability

Data are available from the corresponding author on reasonable request.

## List of abbreviations

HCP: healthcare provider
JIA: juvenile idiopathic arthritis
SH: sexual health
NS: not significant
