# Supplementary material for: Survey of adolescents’ needs and parents’ views on sexual health in juvenile idiopathic arthritis
Source: Pediatr Rheumatol Online J. 2023 Sep 5;21:95. doi: 10.1186/s12969-023-00884-x (PMC10478441; doi:10.1186/s12969-023-00884-x)
Supplement: Supplementary file 2 — Supplementary Material 2 [file 12969_2023_884_MOESM2_ESM.doc]

**Expectations of adolescents (age 10–19 years) regarding sexual health knowledge and communication**

Dear patient,

The Rheumatology Department of the Clermont-Ferrand University Hospital is conducting a study on the expectations of adolescents with juvenile idiopathic arthritis and their parents regarding communication with health professionals in the field of sexual health.

The time period studied is **your care time during your adolescence (age 10 to 19 years). You will need to recall your experiences during that period of your adolescence.**

The results of this survey will help us **propose actions appropriate to this time of life in the field of sexual health.**

The WHO defines sexual health as: “***a state of physical, mental and social well-being in relation to sexuality. It requires a positive and respectful approach to sexuality and sexual relationships, and the possibility of having pleasurable and safe sexual experiences, free from coercion, discrimination and violence.”*** It includes couple relationships, living as a woman and/or man with a chronic illness, countering sexual violence, the prevention of sexually transmitted diseases, bodily pleasure, and reproduction.

This survey involves answering an **anonymous** questionnaire that will take you about 20–25 minutes). Your participation is important, but should you decide not to take part, rest assured that your care will not be affected in any way.

In practical terms, if you agree to take part in this survey, we would like you to answer the following study questionnaire. You do not have to answer all the questions. If you answer the questionnaire, you will be considered as having agreed to take part in the survey.

If you wish, you can obtain further information by email from Carine Savel (csavel@chu-clermontferrand.fr).

Remember that this survey is strictly anonymous. All your personal information will remain confidential. You will be guaranteed right of access and rectification at all times (provided for by the French data protection law of 6 January 1978 (*Informatique et Liberté*), Articles 39 and 40, modified by the Law No. 2004-801 of 6 August 2004), which you can exercise through the Project Leader Carine Savel.

We hope you will agree to take part. Sexuality is often a taboo subject and can make us feel uncomfortable. But sharing your experience will help us take better care of people with idiopathic juvenile arthritis. Thank you.

Sincerely,

**Carine Savel** *(nurse),* Department of Rheumatology, Clermont-Ferrand University Hospital and the Pluridisciplinary Steering Committee, **Sonia Trope**, Director of the National Association for Defence against Rheumatoid Arthritis (ANDAR), **Dr Sandrine Malochet-Guinamond**, rheumatologist at Clermont-Ferrand University Hospital, and **Dr Jean-David Cohen**, rheumatologist at Montpellier University Hospital.

***Some information about you…***

**1.** Gender : 🞏 Male 🞏 Female

**2.** Age: years

**3.** Family status:

🞏 Single 🞏 In a couple 🞏 Divorced/Separated 🞏 Widowed

**4.** Current educational level (whether you are still at school or not)

🞏 Primary 🞏 Junior high school 🞏 High school

🞏 Baccalaureate 🞏 Higher education

**5.** Age at first signs of rheumatism: years

**6.** Age at diagnosis: years

**7.** Type of JIA :

| 🞏 Systemic arthritis or Still’s disease | 🞏 Unclassified arthritis |
| --- | --- |
| 🞏 Oligoarthritis | 🞏 Polyarthritis: 🞏 With rhumatoid factor  🞏 Without rhumatoid factor  🞏 Don’t know |
| 🞏 Arthritis with enthesitis | 🞏 Psoriasic arthritis |
|  🞏 Don’t know |  |

**8.** Are you or have you been a member of an association of patients with JIA?

🞏 Yes 🞏 No

**9.** Are you or have you been in a support network?

🞏 No 🞏 Yes: 🞏 RESRIP (*Réseau Rhumatisme Inflammatoire Pédiatrique*)

🞏 Association KOURIR

🞏 Other (please state) ………………………………………………

**10.** Did you have sexual education lessons at school between 10 and 19 years of age?

🞏 Yes 🞏 No 🞏 I would rather not answer this question

***If YES****: –* how many hours did you have? hours

– How old were you? *(More than one answer allowed)* 🞏 10–15 years 🞏 16–19 years

***Your recollections of adolescence (to age 19 years)…***

**11.** Do you think your rheumatism had an impact on your **love** life (feelings for others, attraction, expression of emotions and feelings, relation with partners, sexual orientation)?

🞏 Yes, a lot 🞏 Yes, a little 🞏 Neither yes nor no 🞏 Not much 🞏 Not at all

🞏 My love life hadn’t started

🞏 Don’t know

🞏 I would rather not answer this question

***If YES****,* for what reasons? (*more than one answer allowed*)

🞏 Body shame

🞏 Reluctance of partners

🞏 Low self-esteem

🞏 Easier for me than for others of my age

🞏 More mature than other adolescents of my age

🞏 Other (please state):……………………………………………………………………...

……………………………………………………………………………………………

……………………………………………………………………………………………

🞏 I would rather not answer this question

**12.** Do you think you had difficulties in your **sex** life in adolescence (concerning sex acts, alone or in a couple)?

🞏 Yes, a lot 🞏 Yes, a little 🞏 Neither yes nor no 🞏 Not much 🞏 Not at all

🞏 My sex life hadn’t started

🞏 Don’t know

🞏 I would rather not answer this question

***If YES***, what difficulties? (*more than one answer allowed*)

| 🞏 Decreased interest in sex | 🞏 Pain during penetration |
| --- | --- |
| 🞏 Increased interest in sex | 🞏 No ejaculation (male) |
| 🞏 Insufficient vaginal lubrication (female) | 🞏 Premature ejaculation (male) |
| 🞏 Weak erection (male) | 🞏 Painful ejaculation (male) |
| 🞏 Late ejaculation (male) | 🞏 Managing pain due to rheumatism |
| 🞏 Tiredness linked to rheumatism | 🞏 Other (please state): …………………….….  …………………………………..…… |
| 🞏 I would rather not answer |  |

**13.** ***If YES***, were these sexual difficulties related to your JIA?

🞏 Yes, a lot 🞏 Yes, a little 🞏 Neither yes nor no 🞏 Not much 🞏 Not at all

🞏 Don’t know

🞏 I would rather not answer this question

**14.** Have you talked with your parents about your **love** life?

🞏 Yes, a lot 🞏 Yes, a little 🞏 Neither yes nor no 🞏 Not much 🞏 Not at all

🞏 Don’t know 🞏 I would rather not answer this question

**15.** Have you talked with your parents about your **sex** life?

🞏 Yes, a lot 🞏 Yes, a little 🞏 Neither yes nor no 🞏 Not much 🞏 Not at all

🞏 Don’t know 🞏 I would rather not answer this question

**16**. Have you ever **shrunk** from broaching the subject of sexual health with a health professional in adolescence?

🞏 Yes 🞏 No

***If YES***, foe what reasons?

………………………………………………………………………………………………………………………………………………………………………………………………………………………………………………………………………………………………………………………………………………………………………………………………………………………………………………………………………………………………………………………………………………………………………………………………………………………………………………………………

***17.*** *What were your sources of information on sexual health in adolescence? (more than one answer allowed)*

| 🞏 Specialized websites | 🞏 Books |
| --- | --- |
| 🞏 Other websites  (please state) ……………………… | 🞏 Teachers  🞏 Health professionals |
| 🞏 Social media (Facebook, Twitter, etc.) | 🞏 Patient associations |
| 🞏 School lessons | 🞏 Information brochures |
| 🞏 Family (parents, brothers and sisters)  (please state) …………………… | 🞏 Radio broadcasts  🞏 TV broadcasts |
| 🞏 Magazines | 🞏 Don’t know |
| 🞏 Films | 🞏 None |
| 🞏 Other (please state) ……….……………...……………….. | 🞏 I would rather not answer this question |

**18.** During your adolescence, was the subject of “***Sexual Health”*** ^(*)^ approached by any health professionals?

🞏 Yes 🞏 No 🞏 Don’t know 🞏 I would rather not answer this question

^(*)^ The WHO defines sexual health as “*a state of physical, mental and social wellbeing regarding sexuality. It requires a postive and respectful approach to sexuality and sexual relationships, and the possibility to have pleasurable and safe sexual experiences free from all coercion, discrimination or violence”.* Sexual health thus goes beyond the sexual act. It includes couple relationships, living as a woman and/or a man with a chronic illness, countering sexual violence, the prevention of sexually transmitted diseases, bodily pleasure, and reproduction.

***If YES, carry on with the questionnaire.***

***If NO or DON’T KNOW*, go straight to Question 22 (Page 7).**

**19.** If the subject of sexual health was approached with a health professional, who broached it first?

🞏 You 🞏 One or both of your parents 🞏 The health professional

🞏 I don’t remember 🞏 I would rather not answer this question

***20.*** *In* ***hospital,*** *with which health professionals did you broach the subject of sexual health? (more than one answer allowed*)

| 🞏 Hospital rheumatologist | 🞏 Occupational therapist | 🞏 Physiotherapist |
| --- | --- | --- |
| 🞏 Hospital pediatrician | 🞏 Nurse | 🞏 Nutritionist |
| 🞏 Doctor of internal medicine | 🞏 Gynecologist | 🞏 Caregiver |
| 🞏 Hospital pharmacist | 🞏 Psychologist | 🞏 Other (please state) |
| 🞏 Child psychiatrist | | …………………………. |
| 🞏 I would rather not answer this question | | |

What were the topics addressed in hospital?

………………………………………………………………………………………………………………………………………………………………………………………………………………………………………………………………………………………………………………………………………………………………………………………………………………………………………………………………………………………………………………………………………………………………………………………………………………………………………………………………………………………………………………………………………………………………………………………………………………………………………………………………………………………………………………………………………………………………………………………………

***21.*** ***OUTSIDE hospital,*** *with which health professionals did you broach the subject of sexual health? (more than one answer allowed)*

| 🞏 Pediatrician | 🞏 Gynecologist | 🞏 Psychologist |
| --- | --- | --- |
| 🞏 School nurse | 🞏 Child psychiatrist | 🞏 Family planning counselor |
| 🞏 General practitioner | 🞏 Rhumatologist | 🞏 Other (please state:………...………....  ……………… ….. |
| 🞏 I would rather not answer this question | | |

What were the topics addressed outside hospital?

………………………………………………………………………………………………………………………………………………………………………………………………………………………………………………………………………………………………………………………………………………………………………………………………………………………………………………………………………………………………………………………………………………………………………………………………………………………………………………………………………………………………………………………………………………………………………………………………………………………………………………………………………………………………………………………………………………………………………………………………

***Ideally…***

**22.** In your opinion, would it have been important to broach the subject of sexual health **while you were receiving hospital care** in your adolescence?

🞏 Yes, very 🞏 Yes, quite 🞏 Neither yes nor no 🞏 Not very 🞏 Not at all

🞏 Don’t know 🞏 I would rather not answer this question

**23.** Would you have liked a health professional to take the first initiative?

🞏 Yes 🞏 No 🞏 Don’t know 🞏 I would rather not answer this question

**24.** In your opinion, **what would be the ideal age** at which health professionals could broach the subject of sexual health in relation to rheumatism in adolescence?

years

🞏 It is not the place of health professionals to broach this subject

🞏 Age does not matter so much as each person’s own experience

🞏 No opinion

🞏 I would rather not answer this question

**25**. In your opinion, what types of **care provision** would be **best suited** to broaching the subject of sexual health with an adolescent? *(more than one answer allowed)*

| 🞏 A routine consultation | If so: | 🞏 In hospital | 🞏 Outside hospital |
| --- | --- | --- | --- |
| 🞏 A consultation dedicated to the subject | If so: | 🞏 In hospital | 🞏 Outside hospital |
| 🞏 A consultation dedicated to the subject, at your child’s request, without you being informed | If so: | 🞏 In hospital | 🞏 Outside hospital |
| 🞏 A Patient Therapeutic Education session^(*)^ | If so: | 🞏 Individually | 🞏 In a group |
| 🞏 Other (please state) …………………………………………………………………….……..  ……………………………………………………………………………  …………………………………………………………………………… | | | |
| 🞏 I would rather not answer this question | | | |

^(*)^ Patient Therapeutic Education aims to help patients acquire or maintain skills they need to best manage life with a chronic illness. It is given in steps and can be offered to individuals or groups.

***26.*** *In your opinion, what would be the* ***most appropriate times*** *in the course of the illness for an adolesecent to broach the subject of sexual health? (more than one answer allowed*)

| 🞏 When the diagnosis is made | 🞏 When side effects occur |
| --- | --- |
| 🞏 At the start of the illness | 🞏 When there is a flare-up |
| 🞏 In remission periods | 🞏 When you request it |
| 🞏 At the start of a new treatment | 🞏 At intervals throughout follow-up |
| 🞏 No particular time | 🞏 I would rather not answer this question |
| 🞏 Other (please state) …………………………  ……………………...…………………… |  |

**27.** I your opinion, which health professionals are the **most competent** to broach this subject with an adolescent? *(more than one answer allowed)*

**In hospital**

| 🞏 Hospital rheumatologist | 🞏 Nurse | 🞏 Physiotherapist | |
| --- | --- | --- | --- |
| 🞏 Hospital pediatrician | 🞏 Occupational therapist | | 🞏 Nutritionist |
| 🞏 Doctor in internal medicine | 🞏 Gynecologist | 🞏 Caregiver | |
| 🞏 Pharmacien(ne) hospitalier | 🞏 Psychologist | 🞏 Adolescent trained to broach the subject of sexual health (“expert”) | |
| 🞏 Other (please state)………...………............................... | | | |
| 🞏 I would rather not answer this question | | | |

**Outside hospital**

| 🞏 Pediatrician | 🞏 Rheumatologist | 🞏 Physiotherapist |
| --- | --- | --- |
| 🞏 Child psychiatrist | 🞏 Sexologist | 🞏 Nutritionist |
| 🞏 Gynecologist | 🞏 Psychologist | 🞏 General practitioner |
| 🞏 School nurse | 🞏 Family planning counselor | 🞏 Patient associations |
| 🞏 Pharmacist | 🞏 Other (please state)………...………............................... | |
| 🞏 I would rather not answer this question | | |

**28.** In your opinion, with which health professional would you have been **most comfortable** broaching the subject? *(more than one answer allowed)*

**In hospital**

| 🞏 Hospital rheumatologist | 🞏 Nurse | 🞏 Physiotherapist |
| --- | --- | --- |
| 🞏 Hospital pediatrician | 🞏 Occupational therapist | 🞏 Nutritionist |
| 🞏 Doctor in internal medicine | 🞏 Gynecologist | 🞏 Caregiver |
| 🞏 Child psychologist  🞏 Hospital pharmacist | 🞏 Psychologist | 🞏 Adolescent trained to broach the subject of sexual health (“expert”) |
| 🞏 Other (please state)………...………............................... | | |
| 🞏 I would rather not answer this question | | |

**Outside hospital**

| 🞏 Pediatrician | 🞏 Rheumatologist | 🞏 Physiotherapist |
| --- | --- | --- |
| 🞏 Child psychiatrist | 🞏 Sexologist | 🞏 Nutritionist |
| 🞏 Gynecologist | 🞏 Psychologist | 🞏 General practitioner |
| 🞏 School nurse | 🞏 Family planning counselor | 🞏 Patient associations |
| 🞏 Pharmacist | 🞏 Other (please state)………...………............................... | |
| 🞏 I would rather not answer this question | | |

**29.** Do you think **opportunities** to communicate on the subject of sexual health with health professionals in hospital are:

🞏 Nonexistent 🞏 Scarce 🞏 Sufficient 🞏 Numerous

🞏 I would rather not answer this question

**30.** In your opinion, what would have helped you **to more easily broach** the subject of sexual health with a health professional **in hospital**? *(more than one answer allowed)*

🞏 The health professional broaching the subject first

🞏 A brochure on the subject being available

🞏 The right occasion (need for contraception, questions on fertility, etc).

🞏 The health professional being comfortable with the subject

🞏 The health professional finding the right occasion

🞏 No parents being present at the consultation

🞏 Having the time

🞏 Being able to talk about it anonymously (via an internet connection)

🞏 Being able to broach the subject with a health professional of the same gender

🞏 Having an informal exchange with another adolescent (while waiting for a consultation or in a hospital room, for example)

🞏 An information video of the subject (that an adolescent can watch)

🞏 A smartphone application (information, follow-up of the illness, etc.)

🞏 I would rather not answer this question

🞏 Other (please state) ……………………….…………………………………..…………

……………………………………………………………………………………….……..

**31.** In your opinion, what would **you have wanted** from health professionals in the field of sexual health? *(more than one answer allowed)*

🞏 Reassurance

🞏 To be listened to

🞏 Discussion

🞏 Guidance to be able to talk with other patients of his or her age

🞏 General information (for example, impact of JIA on sexuality, impact of treatements, choice of contraception, etc.)

🞏 Therapeutic care (for example, lubricants, medication to help erection, psychotherapy, etc.)

🞏 Referral to a specialist if necessary

🞏 I would rather not answer this question

🞏 Other (please state) ……………………….………………………………………..….

……………………………………………………………………………………….……..

***Concerning knowledge of sexual health…***

**32.** In your opinion, what topics would have been useful to address / were actually addressed **by health professionals,** as part of your **hospital** care regarding sexual health?

🞏 I would rather not answer this question

| *Knowledge about* | Yes, certainly | Yes, to some degree | Neither yes nor no | Not really | No, certainly not | **Ideal age to broach the subject** |
| --- | --- | --- | --- | --- | --- | --- |
| **How reproduction works** | | | | | | yrs |
| Topic useful to address | 🞏 | 🞏 | 🞏 | 🞏 | 🞏 |  |
| Topic actually addressed | 🞏 | 🞏 | 🞏 | 🞏 | 🞏 |
| **Fertility and rheumatism** | | | | | | yrs |
| Topic useful to address | 🞏 | 🞏 | 🞏 | 🞏 | 🞏 |  |
| Topic actually addressed | 🞏 | 🞏 | 🞏 | 🞏 | 🞏 |  |
| **Pregnancy and rheumatism** | | | | | | yrs |
| Topic useful to address | 🞏 | 🞏 | 🞏 | 🞏 | 🞏 |  |
| Topic actually addressed | 🞏 | 🞏 | 🞏 | 🞏 | 🞏 |  |
| **Ways in which rheumatic illness is transmitted (heredity, sexual transmission, etc.)** | | | | | | yrs |
| Topic useful to address | 🞏 | 🞏 | 🞏 | 🞏 | 🞏 |  |
| Topic actually addressed | 🞏 | 🞏 | 🞏 | 🞏 | 🞏 |  |
| **How sexuality works (desire, lubrication/erection, orgasm)** | | | | | | yrs |
| Topic useful to address | 🞏 | 🞏 | 🞏 | 🞏 | 🞏 |  |
| Topic actually addressed | 🞏 | 🞏 | 🞏 | 🞏 | 🞏 |  |
| **Impact of rheumatism on sexuality** | | | | | | yrs |
| Topic useful to address | 🞏 | 🞏 | 🞏 | 🞏 | 🞏 |  |
| Topic actually addressed | 🞏 | 🞏 | 🞏 | 🞏 | 🞏 |  |

| *Knowledge about:* | Yes, certainly | Yes, to some degree | Neither yes nor no | Not really | No, certainly not | **Ideal age to broach the subject** |
| --- | --- | --- | --- | --- | --- | --- |
| **What is sexually normal** | | | | | | yrs |
| Topic useful to address | 🞏 | 🞏 | 🞏 | 🞏 | 🞏 |  |
| Topic actually addressed | 🞏 | 🞏 | 🞏 | 🞏 | 🞏 |  |
| **Side effects of treatments for sexuality** | | | | | | yrs |
| Topic useful to address | 🞏 | 🞏 | 🞏 | 🞏 | 🞏 |  |
| Topic actually addressed | 🞏 | 🞏 | 🞏 | 🞏 | 🞏 |  |
| **Definition of different sexual dysfunctions** | | | | | | yrs |
| Topic useful to address | 🞏 | 🞏 | 🞏 | 🞏 | 🞏 |  |
| Topic actually addressed | 🞏 | 🞏 | 🞏 | 🞏 | 🞏 |  |
| **How to treat sexual problems** | | | | | | yrs |
| Topic useful to address | 🞏 | 🞏 | 🞏 | 🞏 | 🞏 |  |
| Topic actually addressed | 🞏 | 🞏 | 🞏 | 🞏 | 🞏 |  |
| **Sexually transmitted diseases and rheumatism** | | | | | | yrs |
| Topic useful to address | 🞏 | 🞏 | 🞏 | 🞏 | 🞏 |  |
| Topic actually addressed | 🞏 | 🞏 | 🞏 | 🞏 | 🞏 |  |
| **Body image and chronic illness** | | | | | | yrs |
| Topic useful to address | 🞏 | 🞏 | 🞏 | 🞏 | 🞏 |  |
| Topic actually addressed | 🞏 | 🞏 | 🞏 | 🞏 | 🞏 |  |
| **Attracting / meeting a partner** | | | | | | yrs |
| Topic useful to address | 🞏 | 🞏 | 🞏 | 🞏 | 🞏 |  |
| Topic actually addressed | 🞏 | 🞏 | 🞏 | 🞏 | 🞏 |  |
| **Learning how to communicate with a partner** | | | | | | yrs |
| Topic useful to address | 🞏 | 🞏 | 🞏 | 🞏 | 🞏 |  |
| Topic actually addressed | 🞏 | 🞏 | 🞏 | 🞏 | 🞏 |  |

| *Knowledge about:* | Yes, certainly | Yes, to some degree | Neither yes nor no | Not really | No, certainly not | **Ideal age to broach the subject** |
| --- | --- | --- | --- | --- | --- | --- |
| **Sexual orientation (homo-, bi-, heterosexuality)** | | | | | | yrs |
| Topic useful to address | 🞏 | 🞏 | 🞏 | 🞏 | 🞏 |  |
| Topic actually addressed | 🞏 | 🞏 | 🞏 | 🞏 | 🞏 |  |
| **Sexual violence – vulnerability – consent** | | | | | | yrs |
| Topic useful to address | 🞏 | 🞏 | 🞏 | 🞏 | 🞏 |  |
| Topic actually addressed | 🞏 | 🞏 | 🞏 | 🞏 | 🞏 |  |
| **Local services and providers for sexual health** | | | | | | yrs |
| Topic useful to address | 🞏 | 🞏 | 🞏 | 🞏 | 🞏 |  |
| Topic actually addressed | 🞏 | 🞏 | 🞏 | 🞏 | 🞏 |  |

What other information do you think would be useful?

………………………………………………………………………………………………………………………………………………………………………………………………………………………………………………………………………………………………………………………………………………………………………………………………………………………………………………………………………………………………………………………………………………………………………………………………………………………………………………………………

Have you any other comments to share on this subject?

………………………………………………………………………………………………………………………………………………………………………………………………………………………………………………………………………………………………………………………………………………………………………………………………………………………………………………………………………………………………………………………………………………………………………………………………………………………………………………………………

***Some last information about you…***

**33.** How many brothers and sisters have you (including stepbrothers and stepsisters)?

**34.** Were your parents separated during your adolescence?

🞏 Yes 🞏 No 🞏 I would rather not answer this question

**35.** As an adolescent, you grew up *(more than one answer allowed)*

🞏 With both your parents

🞏 In a single-parent family

*If so*: 🞏 Mostly with your mother

🞏 Mostly with your father

🞏 The same time with each parent

🞏 In a stepfamily

🞏 Other (please state)………………………………………………………

🞏 I would rather not answer this question

**36.** How old were you when you first had sex (with vaginal or oral penetration)?

years

🞏 I have never had sex

🞏 I would rather not answer this question

***Thank you very much for taking part in this survey!***
